# Ultra-thin flexible rectenna integrated with power management unit for wireless power harvester/charging of smartwatch/wristband

**DOI:** 10.1038/s41598-024-57639-1

**Published:** 2024-03-28

**Authors:** Neeta Singh, Taimoor Khan, Sachin Kumar, Binod Kumar Kanaujia, Hyun Chul Choi, Kang Wook Kim, Karumudi Rambabu, Sembiam R. Rengarajan, Ahmed A. Kishk

**Affiliations:** 1 University School of Automation and Robotics, Guru Gobind Singh Indraprastha University, East Delhi Campus, Delhi, India; 2 Department of Electronics and Communication Engineering, National Institute of Technology Silchar, Silchar, India; 3 Department of Electronics and Communication Engineering, Galgotias College of Engineering and Technology, Greater Noida, Uttar Pradesh India; 4 Department of Electronics and Communication Engineering, Dr. B. R. Ambedkar National Institute of Technology Jalandhar, Jalandhar, India; 5 School of Electronic and Electrical Engineering, Kyungpook National University, Daegu, Republic of Korea; 6 Department of Electrical and Computer Engineering, University of Alberta, Edmonton, Canada; 7 Department of Electrical and Computer Engineering, California State University, Northridge, CA USA; 8 Department of Electrical and Computer Engineering, Concordia University, Montreal, Canada

**Keywords:** Energy science and technology, Engineering

## Abstract

This paper proposes a circularly polarized ultra-thin flexible antenna with a flexible rectifier and power management unit (PMU) for smartwatch/wristband applications. The flexible antenna is compact (0.17*λ*_0_ × 0.20*λ*_0_ × 0.0004*λ*_0_) and has a stepped ground plane. A parasitic element is used at the substrate bottom to reduce the specific absorption rate (SAR) and enhance the gain up to 3.2 dBi, at the resonating frequency of WLAN/Wi-Fi (2.45 GHz). The SAR of the proposed design is also analysed at the resonating frequency, and it satisfies the guidelines of the International Commission on Non-Ionizing Radiation Protection (ICNIRP) and IEEE C95.1–2019 human safety standards. An impedance matching circuit is used between the antenna and the RF energy harvester to improve conversion efficiency. Polarization mismatch is avoided with the help of circular polarization, achieved by tuning stubs of size 0.02*λ*_0_ × 0.044*λ*_0_. The integration of the antenna and rectenna results in a good conversion efficiency of 78.2% at − 5 dBm of input power with a load resistance of 2 KΩ. The availability of RF signals allows the user to charge the smartwatch/wristband by connecting the PMU circuit with the RF energy harvester.

## Introduction

**Figure 1:**
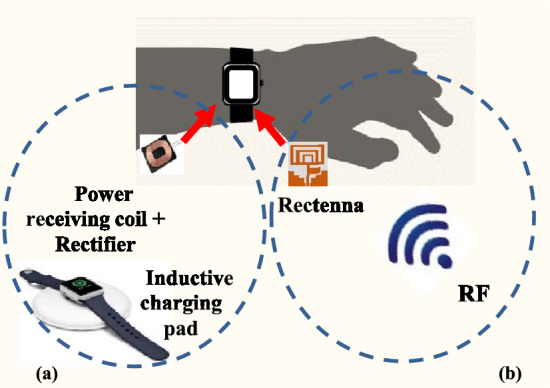
(**a**) The smartwatch/wristband must be removed and placed on the inductive pad for charging, and (**b**) rectenna charges the device via RF energy harvesting.

Globally, tens of millions of people use smartwatches or fitness bands, which can be useful for the early detection of numerous diseases by tracking/measuring people’s temperature, sleeping pattern, heart rate, and travel history^1^. However, a continuous power supply is required for establishing an uninterrupted communication link between the smart device and the base station, as the period of charging or battery change may cause an interruption in data monitoring, reducing accuracy and reliability, as illustrated in Fig. 1^2^. Battery problems are common with portable/wearable devices such as smartphones, smartwatches, fitness trackers, smart glasses, and Bluetooth earbuds/headphones. Energy harvesting could provide a solution to this issue as numerous sources of energy are available in the surroundings^3,4^. There are several types of energy available in the environment, including vibrational (200 µW/cm^2^)^5,6^, thermal (60 µW/cm^2^), pressure, solar (100 mW/cm^2^), and radio frequency (RF) (10 µW/cm^2^)^7,8^. Among these, RF energy harvesting has recently gained more popularity as it is not affected by weather and is continuously available. RF energy harvesting refers to the process of collecting and converting ambient RF radiation into usable electrical energy. RF signals are pervasive in the environment due to the widespread use of wireless technologies such as Wi-Fi, cellular networks, and radio and television broadcasts^9,10^. By harnessing surrounding RF energy, devices can potentially become more energy-efficient and reduce their dependence on conventional power sources. Urban areas with high RF signal density provide an excellent environment for RF energy harvesting. This could be beneficial for smart city applications, where sensors and devices can harness energy from existing communication infrastructure^11,12^.

A power in the range of µW to mW is sufficient to energize the low-power electronic devices. Therefore, the harvested RF energy can provide sufficient power to charge smart wearable devices. An antenna is an important component that collects RF energy, and its size, shape, and orientation play a significant role in RF-to-DC conversion efficiency. The efficiency of the antenna in capturing and converting RF signals into usable electrical energy is affected by its design. A low-profile wearable antenna^13,14^ with high gain, wide-bandwidth, and circular polarization (CP) is required to achieve a high (RF-to-DC) conversion efficiency^15–17^. Also, the wearable device must ensure that the RF energy absorbed by the human body is minimal^18–20^. Some factors to consider when designing wearable gadgets are:

- Wearable devices are worn directly on the body, so comfort is crucial. It should be developed with a lightweight material to ensure that the device is comfortable to wear for an extended period of time.
- Mostly, two types of antennas (flexible and textile) are used for wearable applications. Both flexible and textile antennas contribute to the development of more user-friendly and inconspicuous wearable devices. The choice between them depends on the specific requirements of the application, including the desired level of flexibility and form factor.
- Flexible antennas are typically made from materials that allow them to bend and conform to different shapes without losing their functionality. They are commonly used in applications such as fitness trackers, smartwatches, and other wearable devices that need to conform to the shape of the human body.
- Textile antennas are integrated directly into fabric or clothing materials and are commonly used in smart clothing such as jackets, shirts, or hats. Conductive materials such as metallic threads or conductive inks are often woven or printed into the fabric to create the antenna structure. Since a textile antenna is entirely made of fabric, it shows poor performance.
- The performance of a wearable antenna may degrade due to bending/deformation of the substrate on the body surface. Radiation characteristics and antenna gain may be reduced due to the power absorbed by the human body.
- The specific absorption rate (SAR) should be evaluated to ensure that the power absorbed by the body is negligible or within the guidelines of the International Commission on Non-Ionizing Radiation Protection (ICNIRP) and IEEE C95.1-2019 standards^18^.
- A matching network is used to optimize the impedance matching between the antenna and the energy harvesting circuit. It must be meticulously designed to ensure maximum power transfer from the antenna to the harvesting circuit.

A lightweight high gain flexible material must be used to develop the antenna to meet all of the above-mentioned requirements. Various antenna designs have been presented in the literature that are safe for on-body communication and maintain SAR limits by providing better shielding^21,22^. Flexible antennas have a certain degree of resistance to stretching and bending that does not affect their performance. In Ref.^23^, a pattern-reconfigurable antenna was reported for wearable applications. The antenna offered both broadside and omnidirectional radiation patterns depending on the state of the switching diodes. However, the fabrication of the presented reconfigurable antenna is challenging due to the complexity of integrating RF diodes into the textile material. In Ref.^24^, a cavity slot antenna operating at 2.45 GHz was reported for body area network applications. However, this antenna is not suitable for smartwatch/wristband applications due to its high profile and larger size. In Ref.^25^, a coplanar waveguide (CPW)-fed aperture coupled patch antenna was designed with a reflector to reduce back radiations. The antenna gain was increased by the reflector element. However, this antenna is not suitable for smart devices due to the complex stacked patch architecture and complicated feeding. Metamaterials and ferrite materials, such as artificial magnetic conductors (AMC) and electromagnetic bandgap (EBG), were also used to change the orientation of the electric field in order to reduce back radiations and thus SAR values. In Ref.^26^, a planar Yagi-Uda antenna was designed on a flexible latex substrate and supported by a double AMC layer to improve antenna efficiency and reduce peak SAR. However, the addition of two AMC layers increases the antenna profile, and makes impedance matching difficult, and it also complicates fabrication due to alignment issues with multiple layers. In Ref.^27^, a wearable antenna was presented with an EBG surface, which reduces back radiation and the impact of frequency detuning due to human body losses. However, the used EBG is larger than the antenna size, increasing the size of the wearable device. Additionally, Rohacell foam is used as a spacer between the antenna and the EBG layer to prevent any electrical contacts, which also increases the antenna height and profile.

In this paper, a CPW-fed flexible, ultra-thin circularly polarized antenna with a parasitic element that works as a reflector to enhance gain is proposed. Compared to linear polarization, CP allows for better consumption of the available RF energy, particularly beneficial in real-world scenarios where the orientation of the RF source/harvesting device may vary, as the proposed circularly polarized antenna can capture energy regardless of the incident polarization. The RF power of the antenna is converted to DC power through the process of rectification. The proposed rectifier is also designed on the same substrate material as the antenna in order to realize a flexible rectenna. A matching network and a DC pass filter with a load at the output terminal are also employed to complete the RF-to-DC conversion process. The converter operates in discontinuous conduction mode (DCM) and supplies power irrespective of the user’s movement. The following are the features of the proposed wearable rectenna:

- The proposed rectenna can be seamlessly integrated into smart clothing and accessories due to its low profile, small size, and lightweight, allowing for unobtrusive and efficient energy harvesting. This integration could be particularly useful for health monitoring, fitness tracking, and other smart functionalities.
- Due to the use of a circularly polarized antenna, the presented wearable rectenna can efficiently capture ambient RF signals such as Wi-Fi, cellular, or other electromagnetic signals and convert them into usable electrical power.
- The power converted by the proposed wearable rectenna can be used directly to power a low-power device or it can be stored using supercapacitors. This can lead to a decrease in the environmental impact associated with battery production, usage, and disposal.
- The designed matching network, which is used between the antenna and the rectifier, transfers maximum power by matching the impedance of the antenna with the impedance of the diode.
- In environments with consistent RF signals, the proposed rectenna can provide a continuous and renewable source of power, enabling devices to operate without interruptions. This is particularly beneficial in applications where a constant power supply is critical.

The article is organized as follows: In “Ultra-thin flexible antenna design” section, the antenna design and its development are presented. The rectifier design and its simulation are explained in “Ultra-thin flexible rectifier” section. “Fabrication and experimental results” section describes the bending analysis, SAR assessment, and the parasitic element’s effect. In “Measurement characterization” section, measured results of the antenna and rectenna are presented. A comparative study and conclusion are discussed in “Comparative study” and “Conclusion” sections, respectively.

## Ultra-thin flexible antenna design

This section discusses the proposed antenna structure and its evolution, the concept of CP, and the details of the stepped ground plane.

### Antenna geometry

An antenna is an essential component of wireless communication with desirable characteristics such as high directivity, wide bandwidth, low profile, compact size, low SAR value, and low manufacturing cost. For biomedical applications, the antenna must be resistant to human tissues. To meet all these criteria, various types of antennas have been reported, such as planar inverted F-antenna (PIFA) for ISM-band^28^, the fractal antenna^29^, substrate integrated waveguide (SIW) antenna^30^, Yagi-Uda antenna^31^, and monopole antenna.

**Figure 2:**
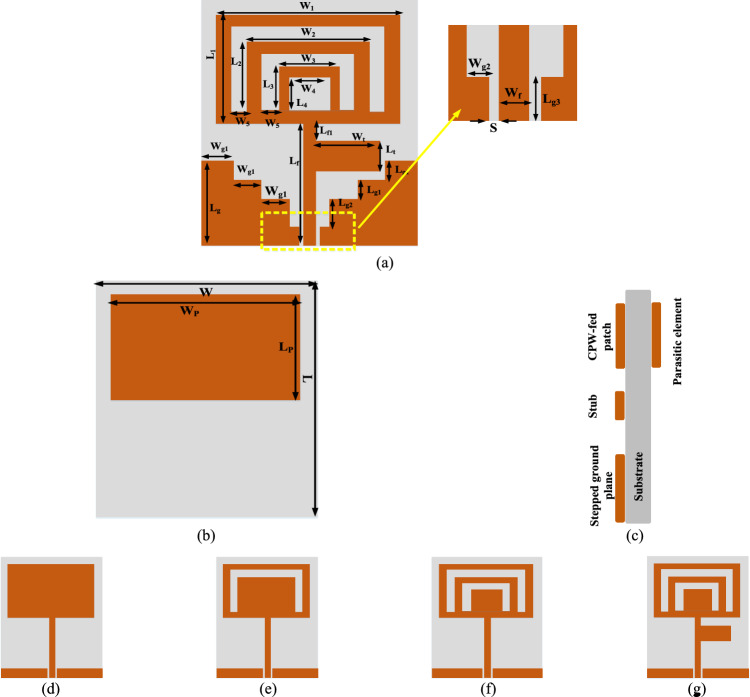
Proposed antenna layout (**a**) front view, (**b**) back view, (**c**) side view (**d**) Antenna 1, (**e**) Antenna 2, (**f**) Antenna 3, and (**g**) Antenna 4.

The proposed antenna geometry is depicted in Fig. 2, and its top and bottom views are depicted in Fig. 2a,b, respectively. The flexible low profile circularly polarized antenna is designed using the full-wave simulation software, ANSYS HFSS. The antenna is fabricated on a low-profile, bio-compatible polyimide substrate of relative permittivity (*ε*_*r*_) of 3.4, loss tangent (tan *δ*) of 0.002, and thickness (*t*) of 0.053 mm. Polyimide material has a low loss factor at high frequencies and a high tensile strength of 165 MPa even at very low thickness, e.g., *t* = 50.8 µm with temperature variations ranging from − 65 to 150 °C^32^. It has a high tolerance to all bending conditions and good electrical properties. Previous research has demonstrated that low profile polyimide substrate has very low dielectric loss when compared to other flexible substrates^33^.

The side view of the antenna is shown in Fig. 2c. The proposed antenna is excited by a CPW feed, which allows for simple surface mount device (SMD) integration with minimal losses. The main radiator is loaded with two U-shaped slots and one square slot, and it is fed by a line of 50 Ω (width (*W*_*f*_) = 1.2 mm and gap (*S*) = 0.1 mm). A parasitic element is used under the radiating patch on the bottom side of the substrate. The parasitic element helps in the suppression of backward radiation without using an extra layer of metamaterial such as AMC, EBG, or reflector^34^. Miniaturization is achieved by using slots, a CPW ground plane, and a tuning stub. The dimensions of the proposed antenna are as follows (in mm): *L* = 25.2, *L*_1_ = 11, *L*_2_ = 7.08, *L*_3_ = 4.34, *L*_4_ = 3, *L*_*f*_ = 12.74, *L*_*t*_ = 6.39, *L*_*g*1_ = 2, *L*_*g*2_ = 3, *L*_*g*3_ = 1.75, *L*_*f*1_ = 1, *W* = 21.5, *W*_1_ = 18.12, *W*_2_ = 12.1, *W*_3_ = 6.1, *W*_4_ = 3.1, *W*_5_ = 1.5, *W*_*t*_ = 6.4, *W*_*g*1_ = 2.85, and *W*_*g*2_ = 1.45.

The design procedure of the CPW-fed antenna is presented in Fig. 2d–g. The characteristic impedance and effective dielectric of the proposed CPW-fed antenna are calculated using Eqs. (1), (2), (3), and (4):

1 $$Z_{0} = \frac{30\pi }{{\sqrt {\varepsilon_{eff} } }}\frac{{K(k^{\prime})}}{K(k)},$$

2 $$\varepsilon_{eff} = 1 + \frac{{\varepsilon_{r} - 1}}{2}\frac{{K(k^{\prime})}}{K(k)}\frac{{K(k_{1} )}}{{K(k_{1}{\prime} )}},$$

where* K* is the elliptic integral of the first order.

$$K = \frac{{W_{f} }}{{W_{f} + 2S}},$$

3 $$k_{1} = \frac{{\sinh \left( {\frac{{W_{f} \pi }}{4t}} \right)}}{{\sinh \left( {\frac{{(W_{f} + 2S)\pi }}{4t}} \right)}},$$

4 $$k^{\prime} = \sqrt {1 - k^{2} } .$$

**Figure 3:**
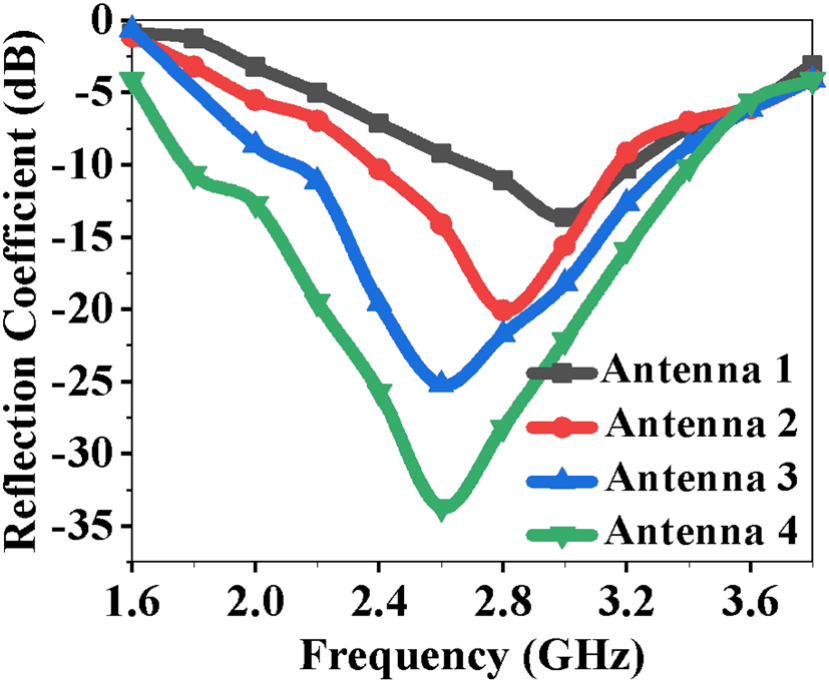
S-parameter comparison of antenna evolution stages.

The reflection coefficients of the antennas shown in the design stages are illustrated in Fig. 3. Antenna 1 (shown in Fig. 2d) is a rectangular patch resonating at 2.8 GHz with − 14 dB reflection coefficient. A U-shaped slot-1 is introduced near the outer edges of the patch to improve antenna performance, as shown in Fig. 2e. This helps to increase the matching bandwidth and shift the resonance towards the left with reflection coefficient of − 20 dB due to the capacitive coupling between the slot and the outermost geometry. Another U-shaped slot (inner slot-2) is introduced at the centre to improve the weak coupling, as shown in Fig. 2f, increasing the matching to − 25 dB and shifting the resonance frequency to 2.6 GHz. A rectangular stub (of size 0.02*λ*_0_ × 0.44*λ*_0_) is loaded near the radiating patch to improve matching and bandwidth, as shown in Fig. 2g. The rectangular stub produces inductive and capacitive effects without changing the resonance frequency, but the bandwidth and matching level are increased to − 32 dB at 2.6 GHz, as shown in Fig. 3.

### Circular polarization

In wireless communication, the receiving antenna must avoid interference regardless of received signal strength or available power. The power received by the antenna is affected by the polarization of the signal. If the polarization changes during transmission due to some obstacle, a small amount of power may be received due to signal fading^35^. This problem can be overcome by using CP. The circular polarized antenna is not affected by Faraday rotation and is less sensitive to multipath interference^36^.

**Figure 4:**
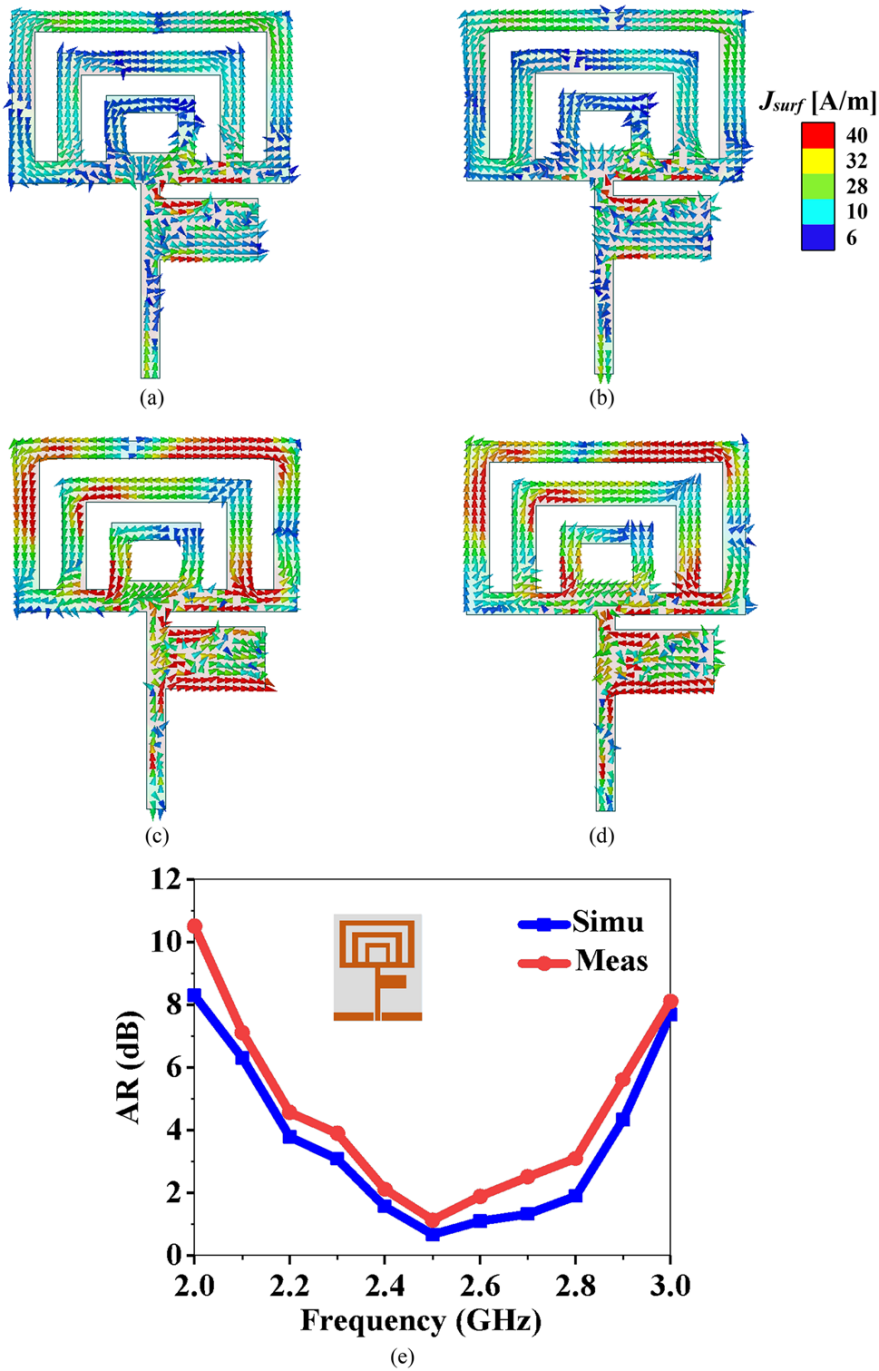
Surface current distribution at 2.45 GHz (**a**) *ωt* = 0°, (**b**) *ωt* = 90°, (**c**) *ωt* = 180°, (**d**) *ωt* = 270°, and (**e**) AR of the proposed flexible antenna (simulated and measured).

The antennas presented in Fig. 2a–g are vertically polarized (linearly polarized). There are various ways to achieve CP. A common method is to truncate the corners of the square patch at ± 45° diagonally^37^, where two orthogonal modes generate, responsible for CP. Asymmetrical cross slot loading is also used to obtain CP^38^, where two modes with the same magnitude and 90° phase shifts are generated. However, both methods are not possible to implement in the proposed antenna structure, therefore a square slot is introduced into the radiating patch, as shown in Fig. 4. Figure 4a–d show the variation of the surface current distribution with time phase at the resonating frequency of 2.45 GHz. At *ωt* = 0°, shown in Fig. 4a, the current distribution is primarily in the *X*-direction, while at *ωt* = 90°, the current is dominant in the *Y*-direction, as shown in Fig. 4b. Figure 4c,d show current vectors of equal magnitude and opposite phase (at *ωt* = 180° and 270°), which are responsible for CP. The electric field vectors from *ωt* = 0° to 270° follows a clockwise path and demonstrates left-hand circular polarization (LHCP) radiation. The square slot produces a suitable phase and magnitude ratio between vertical and horizontal polarization, which improves the CP characteristics as measured by the axial ratio (AR). The simulated and measured AR comparison of the proposed antenna is shown in Fig. 4e.

### Stepped ground plane

**Figure 5:**
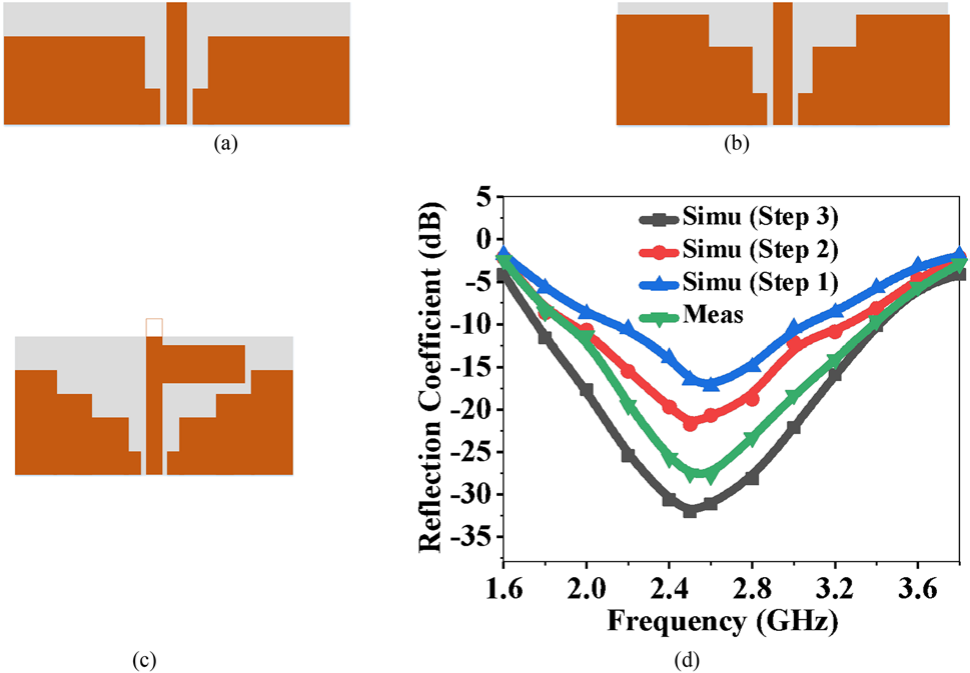
Stepped ground plane of the proposed antenna (**a**) Step-1, (**b**) Step-2, (**c**) Step-3, and (**d**) S-parameters with modifications in the ground plane.

The modification to the ground plane improves impedance matching and bandwidth. Miniaturization is also an important consideration in the design of the RF harvesting antenna. To reduce the resonance frequency to 2.45 GHz, the antenna dimension could be scaled to a larger size. Consequently, the overall size may increase and may not fit inside the watch. The effective length of the antenna may be increased by introducing slots in the ground plane, lowering the resonant frequency. In the proposed antenna, a stepped ground plane is used to avoid increasing the size, shown in Fig. 5. In step-1, as shown in Fig. 5a, the impedance matching at 2.6 GHz decreases. Step-2, shown in Fig. 5b, illustrates a minor shift in resonance at 2.55 GHz. In step-3, as displayed in Fig. 5c, the coupling improves, resulting in an increase in impedance matching and a shift to a resonant frequency of 2.45 GHz. Figure 5d depicts the variation in reflection coefficients at the 1-, 2-, and 3-step stepped ground planes.

## Ultra-thin flexible rectifier

A rectenna is used to harvest freely available RF energy from the ambient environment. Using energy harvesting technology, a rectenna can either replace or recharge the battery. Rectenna senses RF power using an antenna and converts it into DC power through the rectification process. In order to realize a flexible rectenna, the rectifier is also designed using the same substrate material (*ε*_*r*_ = 3.4, tan *δ* = 0.002, and *t* = 0.05 mm) as the antenna. It consists of a matching network, a rectifying circuit, and a DC pass filter with a load at the output terminal. In the literature, various rectifier topologies have been studied to select the most suitable circuit. A few parameters must be considered before selecting the topology and diode. The circuit must be compact, therefore, miniaturization is needed. Second, the reflection coefficient of the rectifier for 50 Ω impedance should be minimized, and third, RF-to-DC conversion efficiency should be maximized^39–41^.

Filters play an essential role in RF energy harvesting circuits, helping to optimize energy extraction efficiency, reduce noise and interference, ensure regulatory compliance, and enhance signal quality^42,43^. The enhanced signal quality leads to more stable and reliable operation of downstream components such as rectifiers and energy storage devices, ultimately improving the overall performance of the energy harvesting system^44,45^. The proper design and integration of filters are critical to the successful implementation of RF energy harvesting systems.

### Flexible rectifier circuit

**Figure 6:**
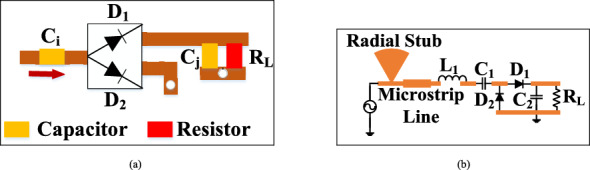
(**a**) Schematic of the rectifier, and (**b**) schematic of the diode.

A minimum circuitry is required at a low input power level (≈ 0 dBm) for wearable applications and the need for a flexible circuit. The ambient power available in the environment is low (− 50 dBm to 0 dBm). Obtaining a high efficiency at such a low input power level is a challenge for the rectifier circuit. Various topologies have been studied and analysed, such as a half-wave rectifier, a full-wave rectifier, and the voltage doubler. Voltage multipliers (doubler) increase the voltage level by adding diode and capacitors. The capacitors function as a storage element. Voltage multipliers ranges from one to *n* stages. The proposed design employs a voltage doubler to meet the minimum circuitry requirement, as shown in Fig. 6a. The proposed voltage doubler consists of two diodes and two capacitors connected in series. The diode-capacitor combination doubles the sinusoidal input voltage, and the component values are *L*_1_ = 6 nH, *C*_1_ = 100 pF, and *C*_2_ = 36 pF. The load resistance *R*_*L*_ is a part of the rectifier circuit. The output DC voltage measured across the load resistance of 2 KΩ is optimized to achieve high efficiency.

### Simulation of the rectifier

The flexible rectifier is designed using the Keysight ADS software. The proposed design is simulated using a harmonic balance (HB) to evaluate conversion efficiency at different power levels, while scattering parameter (SP) controlled is used to estimate the S-parameter of the rectifier. The maximum output DC voltage is realized at 2 kΩ for the input power level of − 5 dBm. The selection of an RF diode is important in order to obtain the optimum output voltage with high efficiency. The RF diode must be capable of rectifying the low input RF power that is freely available in the environment and have a low turn-on voltage. Among all microwave diodes, Schottky diodes exhibit all the characteristics required for RF energy harvesting. Therefore, in this work, HSMS-282E in a SOT 323 package is chosen, which is a common anode diode package. It has a low turn-on voltage of around 0.34 V and a low series resistance (*R*_*S*_ = 6 Ω) for high power dissipation, resulting in better thermal conductivity. Figure 6a shows the circuit diagram of the common anode diode, HSMS-282E. The parameters of the diode equivalent circuit are: *C*_*J*0_ = 0.7 pF, *V*_*B*_ = 15 V, and *E*_*G*_ = 0.69 eV. The diode current can be evaluated as

5 $${I}_{D} = {I}_{S}\left[{\text{exp}}\left(\frac{a{\text{sin}}\omega t-0.5{V}_{DC}}{m{V}_{T}}\right)\right],$$

where *I*_*D*_ is the diode current, *V*_*DC*_ is the output DC voltage, and *I*_*S*_ is the saturation current. A matching network is used between the antenna and the rectifier to transfer maximum power by matching the impedance of the antenna with the impedance of the diode.

### High impedance matching circuit

At high frequencies, various impedance matching methodologies, such as tuning stubs, lumped circuits, and quarter-wave transformers, have been investigated. Quarter wave transformers only match real impedances. The lumped element matching technique is found unsuitable at high frequencies due to unpredictable parasitic and narrow bandwidth performance. Tuning stubs are the best choice for achieving wide bandwidth with a complex matching network. Also, the impedance matching circuit should be compact. Radial tuning stubs offer a wider bandwidth and require less space at higher frequencies than rectangular tuning stubs. The employed radial stub has a width of 1.65 mm, a length of 1.6 mm, and an angle of 35°, and a microstrip line with a length of 1.8 mm and a width of 0.9 mm is used, as shown in Fig. 6b. The amount of power may increase or decrease depending on the availability of ambient RF energy. The power management unit (PMU) stabilizes the system and stores power for later use.

### Power management unit (PMU)

**Figure 7:**
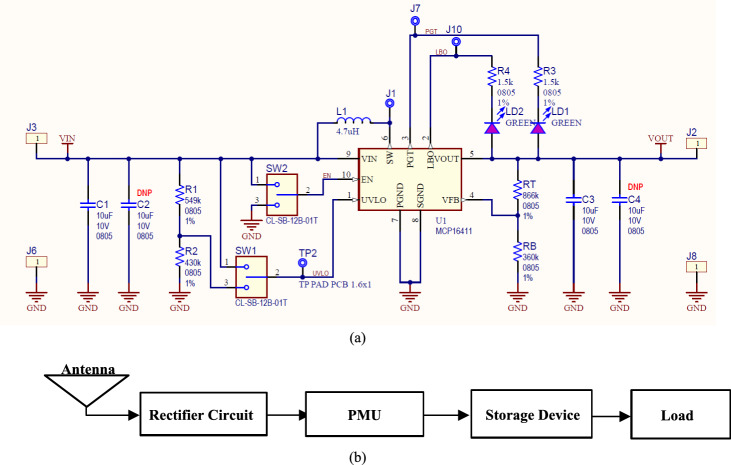
Boost convertor circuit (**a**) schematic, and (**b**) power management unit with the rectenna block diagram.

The PMU, which consists of a boost converter and an oscillator, is an important component for optimizing the conversion efficiency of the rectenna. The boost converter is known as a step-up converter, as it increases the voltage level from input to output, shown in Fig. 7a. At the load, supercapacitors are used as a storage element to store the voltage to be used in the discontinuous conduction mode (DCM). The PMU must maintain the same voltage level at varying input power levels, as shown in Fig. 7b. However, the power level may vary from one place to another due to varying RF energy sources available.

The boost converter provides stored energy for charging to overcome the discontinuity problem when charging the smartwatch/wristband. The input impedance can be calculated using Eq. (6)

6 $${R}_{B}=\frac{2{L}_{B}{F}_{S}}{D}\left(1-\frac{{V}_{R}}{{V}_{B}}\right),$$

where *L*_*B*_ is the boost inductance, *F*_*S*_ is the switching frequency of a switching device, which can be a MOSFET, BJT, PIN diode, or Schottky diode, and *D* is the duty cycle of the oscillator used to control the boost convertor. *V*_*R*_ and *V*_*B*_ are the voltages of the rectifier and boost circuits, respectively^46–48^.

## Fabrication and experimental results

**Figure 8:**
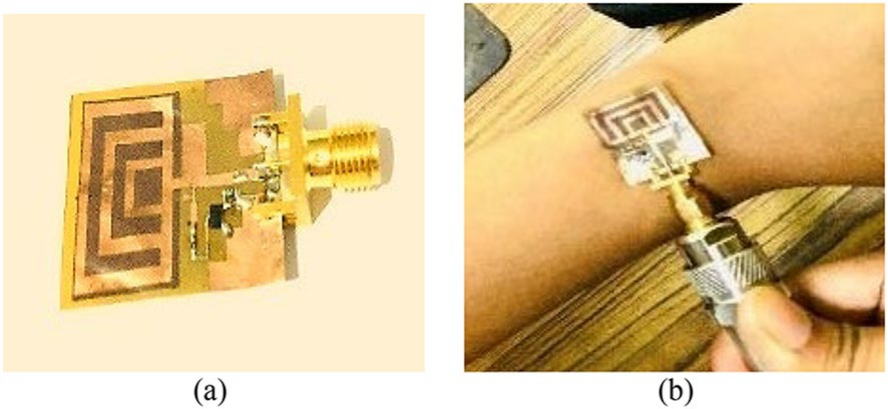
Smartwatch rectenna (**a**) In free space, and (**b**) on the wrist.

A prototype with the PMU module is fabricated and tested to validate the proposed flexible ultra-thin RF energy harvesting system. A network analyzer is used to measure the S-parameters in free space and on the wrist. Figure 8a,b show the fabricated rectenna for wireless charging of a smartwatch.

**Figure 9:**
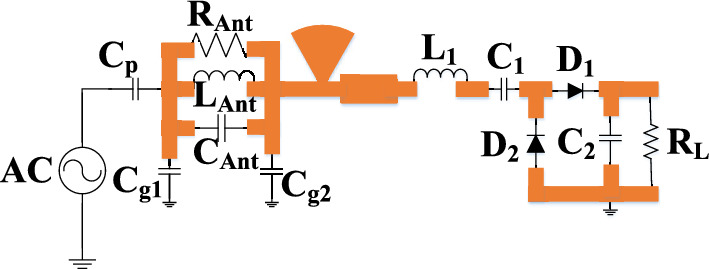
Equivalent circuit of the antenna with the rectifier circuit.

The impedance matching circuit matches the antenna impedance with the rectifier to improve conversion efficiency. The *RLC* resonant circuit has been widely used to represent the radiating patch near the resonating frequency. The proposed equivalent circuit, shown in Fig. 9, is a modified circuit of the antenna. The equivalent circuit is a parallel *RLC* resonator operating at 2.45 GHz with parallel capacitors for ground. The equivalent circuit is directly connected to the rectifier circuit via an impedance matching circuit to offer a perfect match.

**Figure 10:**
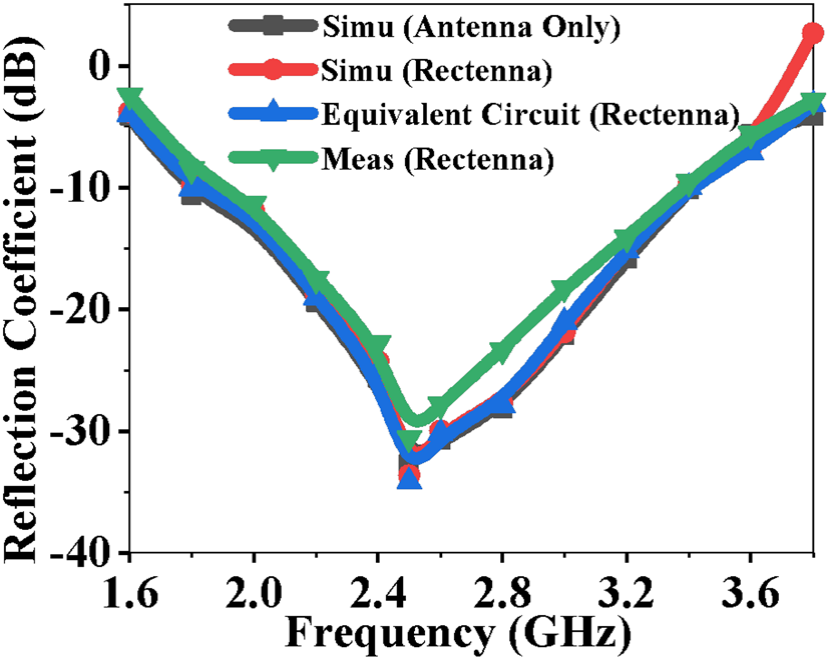
Rectenna performance comparison (simulated, measured, and equivalent circuit results).

Figure 10 shows the reflection coefficients of the antenna with/without the rectifier, the equivalent circuit of the rectenna, and measured results. The admittance of the antenna circuit is

7 $${Y}_{0}=\frac{1}{{R}_{Ant}+\omega {L}_{Ant}+\frac{1}{\omega {C}_{Ant}}}+j\left(\omega {C}_{g1}- \omega {C}_{g2}\right).$$

Here, the real part of the admittance is

8 $${\left(Real\right) Y}_{0}=\frac{{R}_{1}}{{L}_{Ant}^{2}{\left(\frac{1}{\omega {{L}_{Ant}C}_{Ant}}-\omega \right)}^{2}+{R}_{Ant}^{2}},$$

and the imaginary part of the admittance is

9 $${\left(Img\right) Y}_{0}=\frac{{L}_{Ant {\left(\frac{1}{\omega {{L}_{Ant}C}_{Ant}}-\omega \right)}^{2}}}{{L}_{Ant}^{2}{\left(\frac{1}{\omega {{L}_{Ant}C}_{Ant}}-\omega \right)}^{2}+{R}_{Ant}^{2}}+ \left(\omega {C}_{g1}- \omega {C}_{g2}\right).$$

### Bending analysis

**Figure 11:**
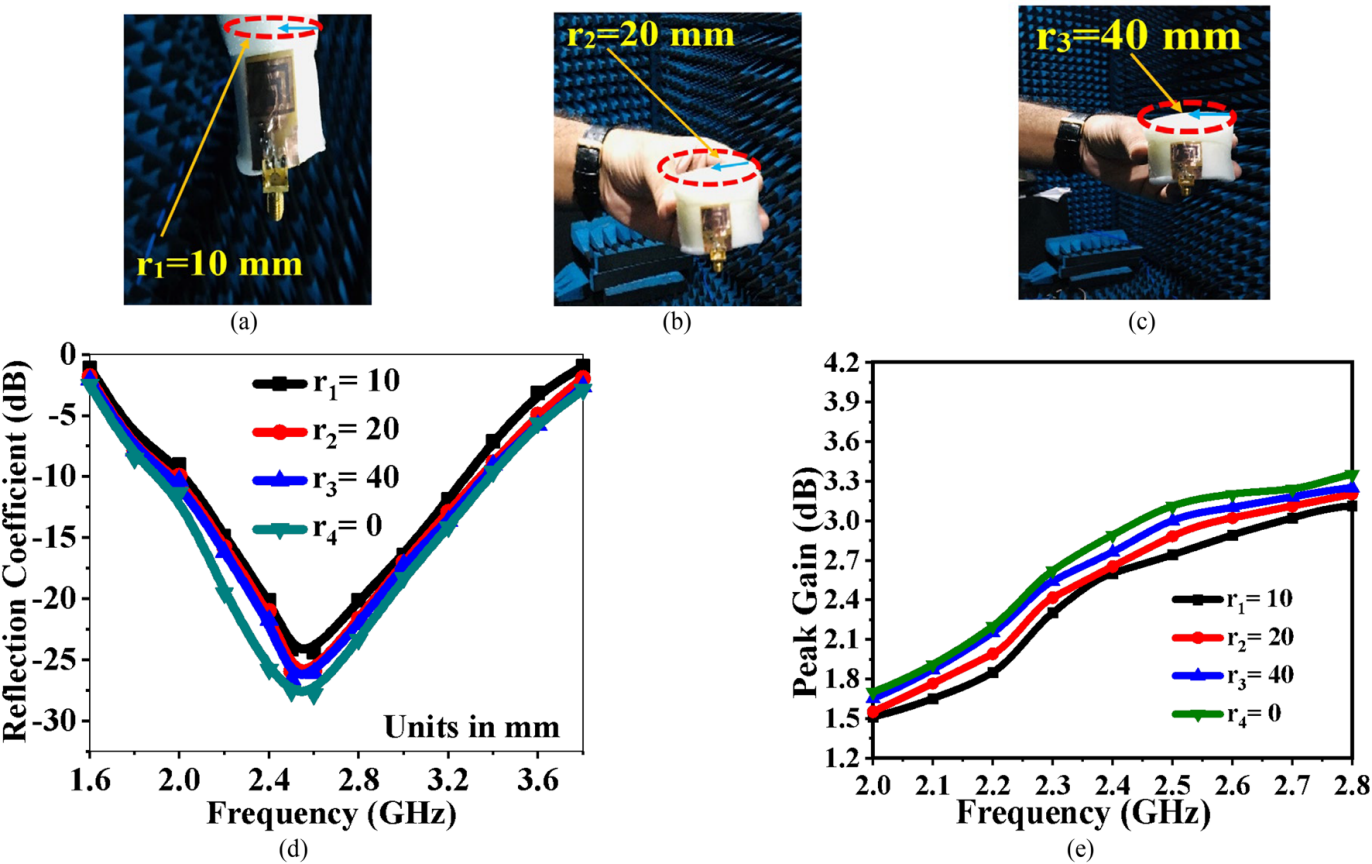
Fabricated rectenna with different bending radii (**a**) *r*_1_ = 10 mm, (**b**) *r*_2_ = 20 mm, (**c**) *r*_3_ = 40 mm, (**d**) S-parameters at different bending radii, and (**e**) Gain variation at different bending radii.

The bending performance of the wearable rectenna must be evaluated to ensure that the device can work effectively when deformed. The bending effect of the smartwatch rectenna is investigated for three different radii along the *X*- and *Y*-axes. Figure 11 shows the fabricated rectenna bent over a foam cylinder with a dielectric constant of 1.03. Figure 11a–c depict the different radius of a foam cylinder (10 mm, 20 mm, and 40 mm, respectively). The S-parameters for all three bent cases are shown in Fig. 11d. The bending effect results in impedance mismatch, and thus the resonant frequency shifts towards the lower side, but no significant change in bandwidth is observed. The performance of the rectenna is not significantly affected by its compact size. Before bending, the reflection coefficient bandwidth is 1.43 GHz (1.78–3.21 GHz). The reflection coefficient bandwidth is reduced by a small amount to 1.24 GHz (1.68–2.92 GHz) for bending radius *r*_1_ = 20 mm. Figure 11e depicts the variation of gain at various radii, but no significant loss in gain is observed.

### Specific absorption rate (SAR) analysis

**Figure 12:**
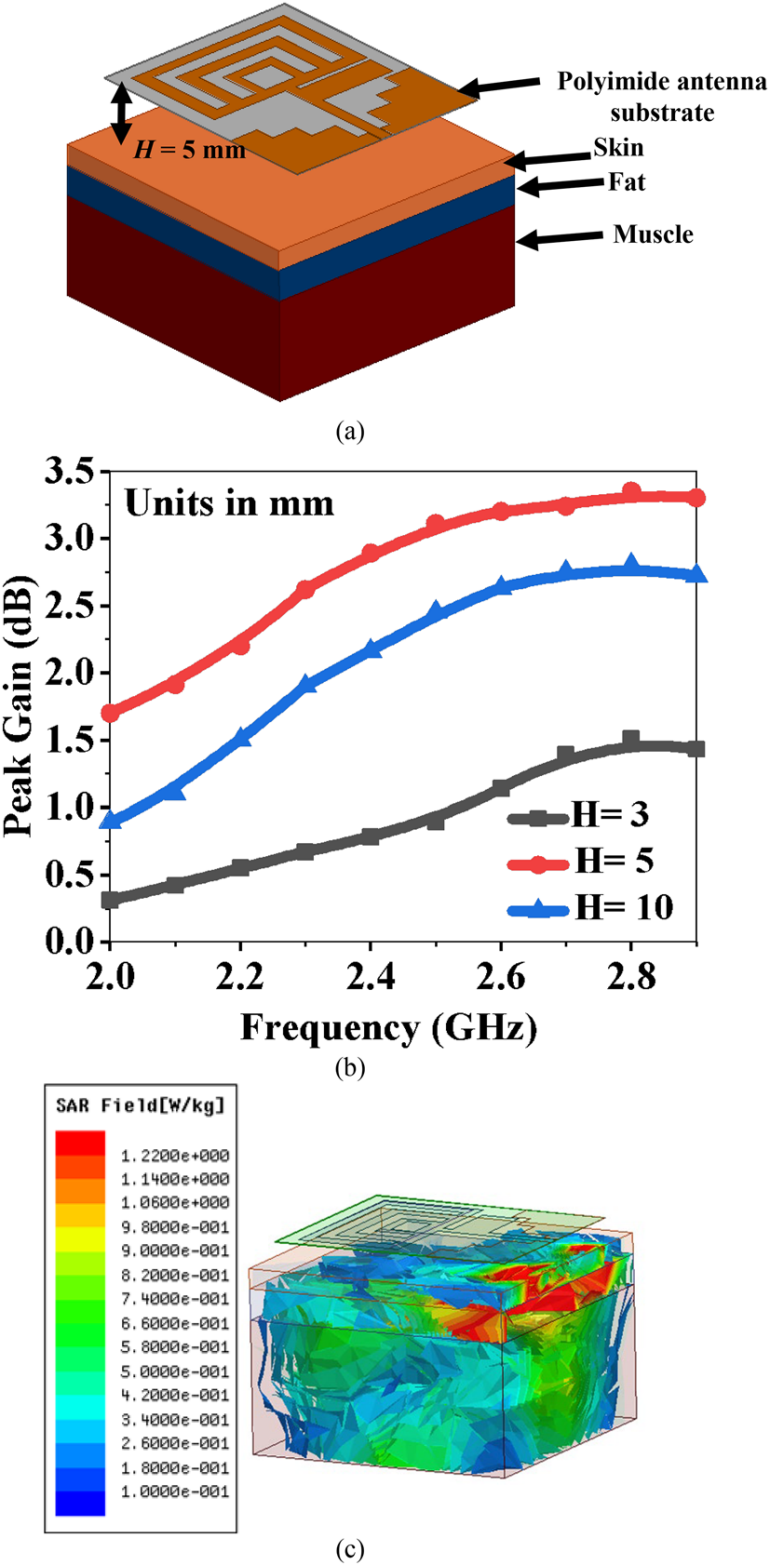
(**a**) Antenna with hand tissue model, (**b**) gain variation at different *H*, and (**c**) SAR distribution at *H* = 5 mm.

SAR is an important antenna characteristic for wearable applications. SAR is the rate at which the human body absorbs energy in the presence of EM radiation, and it can be evaluated using Eq. (10).

10 $$SAR=\frac{\sigma {E}^{2}}{\rho },$$

where $\rho$ is the mass density, *E* is the intensity of the electric field, and $\sigma$ is the conductivity of human tissue. Figure 12a shows the SAR analysis in the human tissue model when the antenna is 5 mm away from the human tissue.

Figure 12a shows that the tissue model consists of three layers: (1) a skin layer of thickness of 3 mm with *ε*_*r*_ = 38.6 and *σ* = 3.71 S/m, (2) a fat layer of thickness of 4 mm with *ε*_*r*_ = 5.9 and *σ* = 0.11 S/m, and (3) a muscle layer of thickness of 13 mm with *ε*_*r*_ = 52.7 and *σ* = 1.71 S/m. Figure 12b shows the reflection coefficients at various distances between the antenna and the tissue. Simulated SAR values at a 5 mm distance are 1.22 W/kg for 20 dBm and 0.986 W/kg for 10 dBm, as shown in Fig. 12c. The simulated SAR values are within the IEEE standard limit of less than 1.6 W/kg for 1 g of tissue.

**Figure 13:**
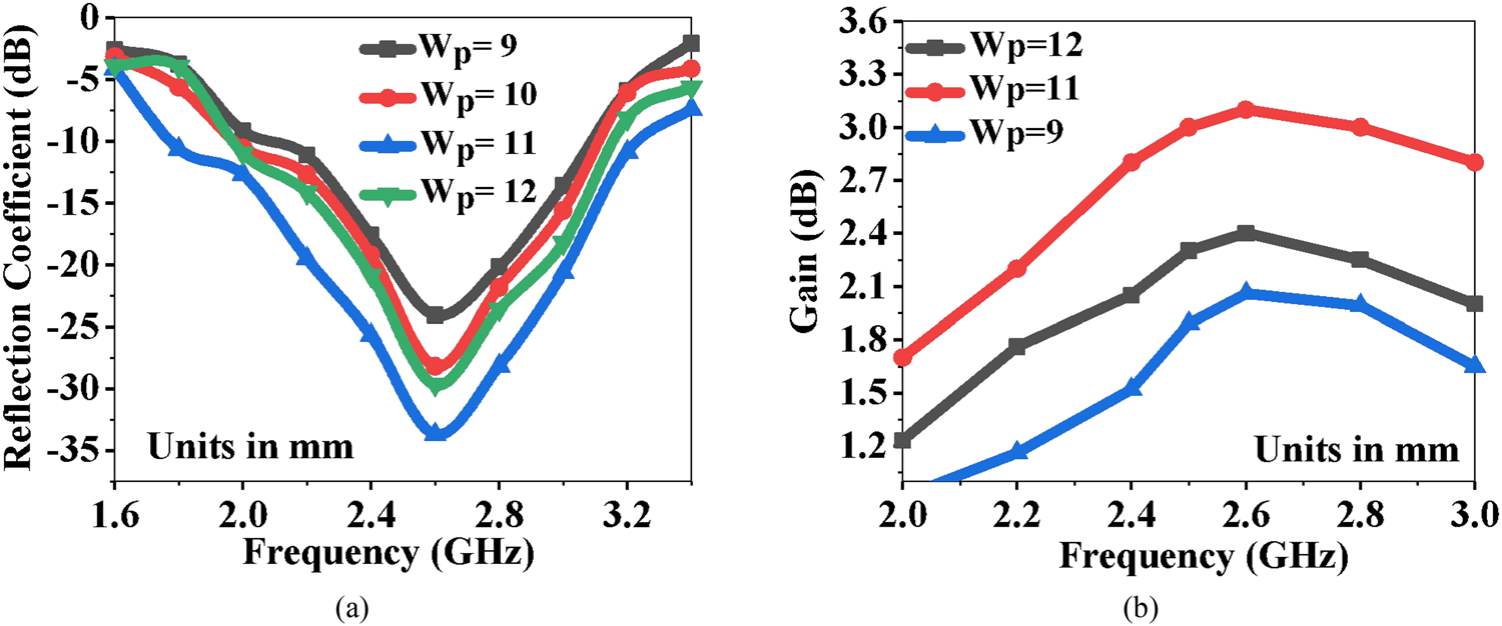
Variations in antenna performance due to the parasitic element (**a**) reflection coefficients, and (**b**) peak gain.

The parasitic element is working as a reflector on the bottom side of the antenna as it is not connected to the common ground plane. It is used as a buffer between the antenna and the human tissues to reduce the EM waves impinging on the human tissues. Since it is underneath the radiating patch and close to the edges of the substrate, it can also suppress surface waves. Surface waves reduce gain and radiation efficiency, and hence, the parasitic element is also responsible for increasing gain. Figure 13a shows the effect of varying the width (*W*_*P*_) of the reflector. The best results are obtained at the same size as the patch width of the antenna.

**Figure 14:**
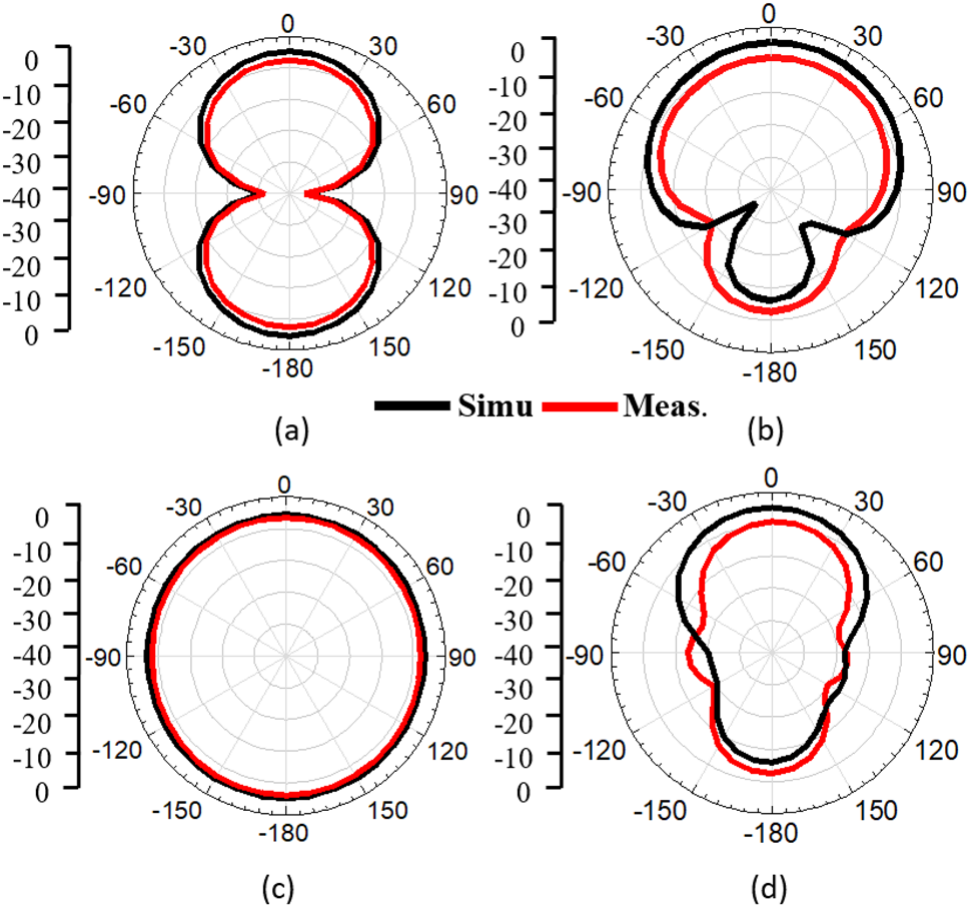
Radiation patterns of the antenna on human tissue (**a**) without the parasitic element at E-plane (*XZ*-plane), (**b**) with the parasitic element at E-plane (*XZ*-plane), (**c**) without the parasitic element at H-plane (*YZ*-plane), and (**d**) with the parasitic element at H-plane (*YZ*-plane).

Also, backward radiation must be reduced in order to keep the SAR within the specified range. The methods employed in the literature, such as using an AMC, cavity-backing, and a large reflector, are effective, but they increase the height and the size of the structure, which is not desirable. In the proposed design, a bio-compatible substrate is used with the reflector to reduce SAR while achieving miniaturization. Figure 13b shows the variation in gain with varying reflector widths. The simulated and measured radiation patterns with/without reflector are shown in Fig. 14a–d. The radiation patterns are measured in an anechoic chamber by rotating 5° every time to cover − 180° to + 180° range.

## Measurement characterization

### Antenna measurement

**Figure 15:**
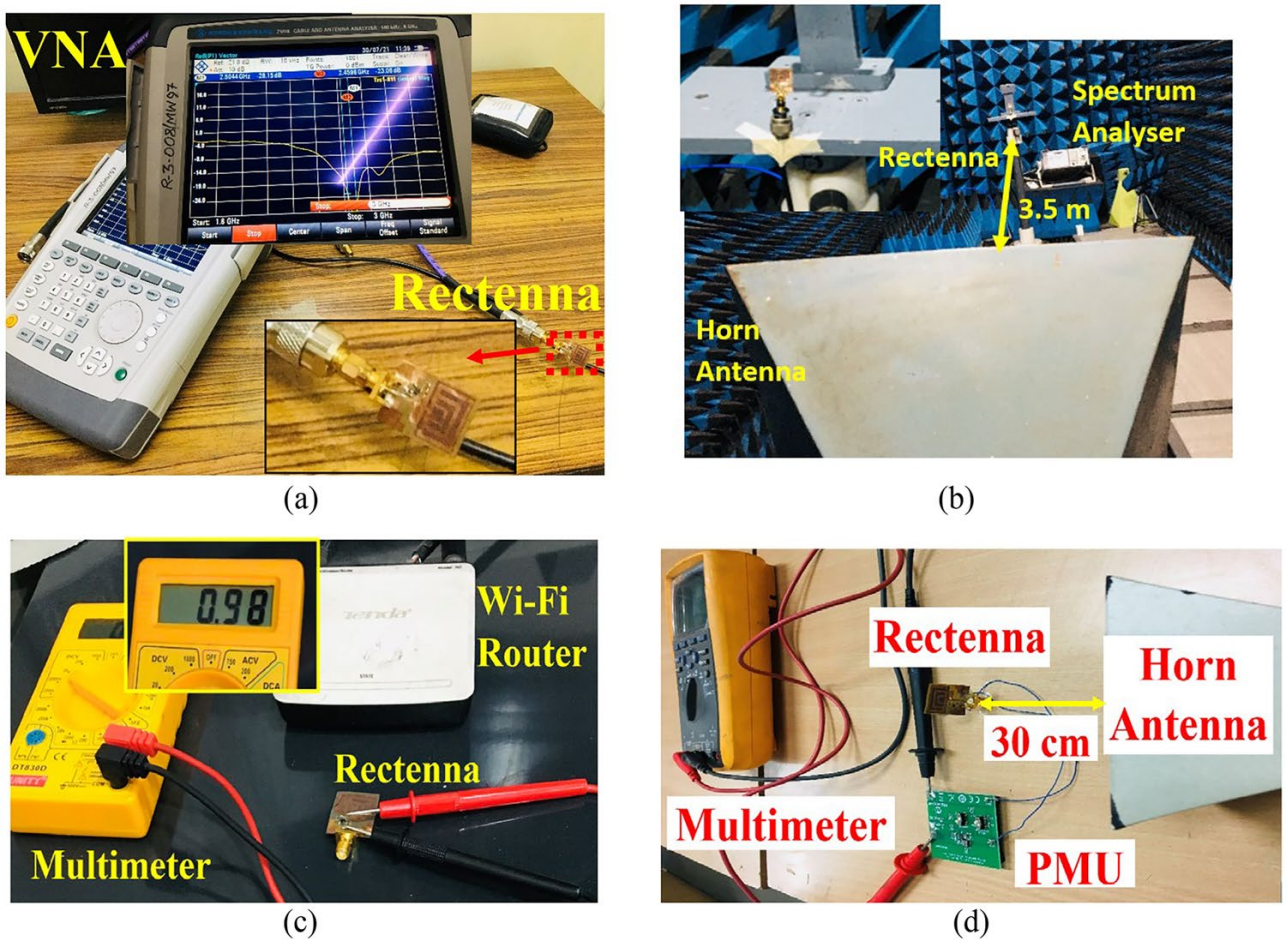
Measurement set-up of the rectenna under test (RUT) in an anechoic chamber (**a**) layout, (**b**) wireless test set-up, (**c**) rectenna measurement in an indoor environment, and (**d**) rectenna measurement with a PMU module.

Figure 15a shows the antenna prototype measurement using the vector network analyser (VNA). The far-field radiation of the antenna (at 2.4 GHz) measured in an anechoic chamber is shown in Fig. 15b. When the antenna is placed on the human wrist with a smartwatch prototype, the resonant frequency shifts to a higher frequency of 2.5 GHz. This is because the matching impedance of the antenna that was matched without the presence of human tissue becomes mismatched with a detuned resonant frequency in the presence of human tissue. However, as the impedance bandwidth is wide, the frequency detuning results in a minor shift.

### Rectenna measurement

As shown in Fig. 15b, a horn antenna with a high gain in the frequency range of 1 to 18 GHz is used as a transmitting antenna. The horn antenna is connected to a signal generator via a connector cable. The proposed rectenna is placed in the anechoic chamber at a 3.5 m distance from the horn aperture. The power received by the receiving flexible antenna is measured using a spectrum analyser.

The measured signal may vary from the simulated results due to cable loss, and the output DC voltage is measured across a load resistor *R*_*L*_ = 2 KΩ with a multimeter, as shown in Fig. 15c. The Friss transmission Eq. (11) is used to calculate the received power

11 $${P}_{r }= {\left(\frac{\lambda }{4\pi r}\right)}^{2}{P}_{t}{G}_{t}{G}_{r},$$

where *P*_*t*_ is the transmitted power, *G*_*t*_ is the gain of the transmitting (horn) antenna, *G*_*r*_ is the gain of the receiving antenna (flexible bio-compatible), and *r* is the distance between the horn antenna and the proposed antenna. Figure 15c shows the measurement setup of the antenna embedded rectifier circuit in the indoor environment. The Wi-Fi router serves as the RF source, and the RF signal is collected and converted into DC power by the proposed rectenna. The available output voltage in the indoor environment is 0.98 V, which is less than the output voltage in the outdoor environment, i.e., 2.46 V. Figure 15d shows the measurement set up of the RF energy harvesting system with the PMU to boost the input voltage and store it for later use. The boost converter can boost the input voltage of range (from 0.8 to 5.5 V) to 3.3 V fixed.

**Figure 16:**
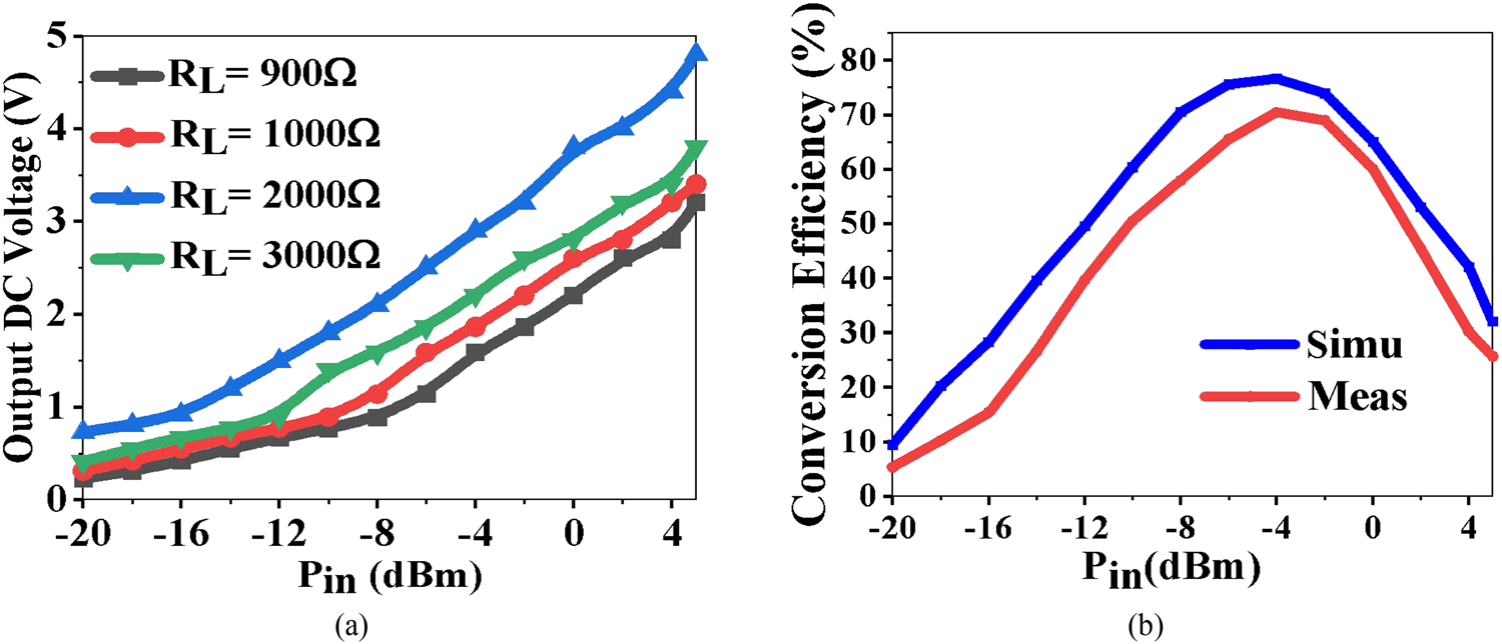
Rectenna characteristics (**a**) output DC voltage, and (**b**) conversion efficiency.

Figure 16 shows the simulated and measured efficiency of the rectenna at 2.45 GHz, with varying input power levels.

12 $${\eta }_{CE}=\frac{{P}_{DC}}{{P}_{in}}\times 100\%=\frac{{{V}^{2}}_{DC}}{{R}_{L}{P}_{in}} \times 100\%,$$

13 $${P}_{DC}= \frac{{V}_{DC}^{2}}{{R}_{L}}.$$

**Figure 17:**
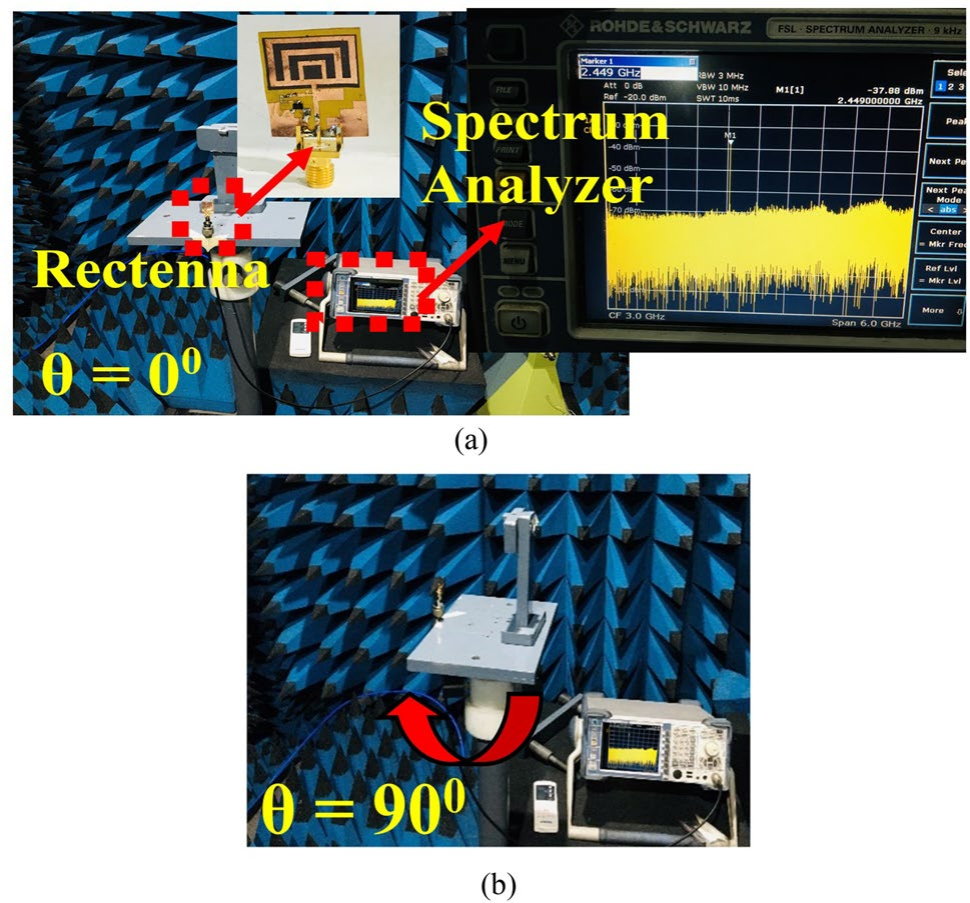
Anechoic chamber setting (**a**) received power at *XZ*-plane (*θ* = 0°), and (**b**) *YZ*-plane (*θ* = 90°).

The output DC power is calculated using Fig. 16a and Eq. (12). The overall performance of the antenna and rectifier decides the conversion efficiency. The conversion efficiency of 78.2% is a maximum at − 5 dBm, as shown in Fig. 16b. Figure 17 shows the measurement of the rectenna using the spectrum analyzer. In Fig. 17a, − 38 dBm power is received at 2.45 GHz at *θ* = 0°, while in Fig. 17b, the plane is rotated by 90°.

## Comparative study

The flexible circularly polarized antenna is compared with other reported flexible and non-flexible single-band antennas. Wearable applications require a small size antenna. Table 1 compares the proposed antenna design with previously published works that used different technologies to achieve high gain and miniaturization. It is evident that the proposed antenna is compact, robust, low profile, and circularly polarized with a good amount of gain in the presence of a human wrist. Table 2 compares the proposed rectenna characteristics with previously published works. The proposed rectenna is flexible and low profile, with good conversion efficiency and output DC voltage. The following are the main advantages of the proposed rectenna:

- The proposed rectenna operates at the 2.45 GHz frequency (ISM-band), which is the most used frequency in wireless applications/systems, allowing to collect more RF energy than previously reported rectennas^31,41,47,48^.
- In contrast to the antennas reported in Refs.^19,20,23^, which used complex configurations such as reconfigurability, EBG, and HIS, the proposed antenna uses a slotted patch, a stepped ground plane, and a simple reflector.
- The proposed rectenna employs a CPW-fed antenna, which offers a wide bandwidth and easier integration with monolithic microwave integrated circuits due to the use of a single metallic layer on one side of the substrate.
- In comparison to the antennas reported in^13,19,20,23,29^, the proposed flexible antenna occupies less chip space while exhibiting a high gain.
- Unlike the rectenna configurations^31,40,41^, the proposed rectenna has a high output voltage.
- The rectennas reported in^31,40,41,47,48^, were designed on rigid dielectric materials, whereas the proposed rectenna is fabricated on a flexible substrate.

**Table 1:** Comparison of proposed antenna with previously reported works.

Ref.	Frequency (GHz)	Gain (dBi)	Overall size (mm^2^)	Antenna type	Antenna substrate	Flexible
^13^	2.44	6.6	115 × 73	Probe feed patch	Woven polyester	Yes
^19^	2.45	4.5	38.75 × 20	AMC-backed antenna	Kapton polyimide	Yes
^20^	2.40	− 5	38 × 38	HIS antenna	FR-4	No
^23^	2.45	2 to 3.9	100 × 100	Reconfigurable patch	Felt	No
^39^	2.45	5.6	35.2 × 33.4	CPW-fed with slots	FR-4	No
Prop	2.45	3.2	21.5 × 25.5	CPW-fed patch	Kapton polyimide	Yes

**Table 2:** Comparison of proposed rectenna with previously reported works.

Ref.	Frequency (GHz)	*P*_*in*_ (dBm)	Conversion efficiency (%)	Diode	*V*_*out*_ (V)	Flexible	CP
^31^	2.14	− 20	50	HSMS-2852	0.484	No	No
^40^	2.45	0	65.1	SMS-630-005 LF	1.65	No	Yes
^41^	3.5, 5.8	5	54.53	HSMS-2860	1.31	No	Yes
^47^	0.915	10.4	88	HSMS-286C	5.2	No	No
^48^	0.905	4.18	90.6	HSMS-286C	4.18	No	Yes
Prop	2.45	− 5	78.2	HSMS-282E	2.46	Yes	Yes

## Conclusion

In this paper, a circularly polarized flexible rectenna is presented with good conversion efficiency and output DC voltage. The designed rectenna is less sensitive to bending and deformation because of its wide bandwidth and use of the flexible substrate. The antenna structure is designed, built, and tested under different conditions, including bending/deformations and human tissues. The proposed CPW-fed flexible antenna has a broad bandwidth due to the stepped ground plane. A tuning stub is used to obtain CP, which is an important specification for wireless communication systems. Also, a parasitic element is used to reflect and direct the beam in the forward direction, thereby enhancing gain. The rectifier is designed on the same substrate as the antenna, maintaining a miniaturization and impedance matching circuit for bio-compatible substrate material, and antenna and rectenna integration results in good conversion efficiency of 78.2% at − 5 dBm of input power.

## Data Availability

The datasets used and/or analysed during the current study available from the corresponding author on reasonable request.

## References

[1] Yakoh A, Pimpitak U, Rengpipat S, Hirankarn N, Chailapakul O, Chaiyo S (2021). Paper-based electrochemical biosensor for diagnosing COVID-19: Detection of SARS-CoV-2 antibodies and antigen. Biosens. Bioelectron..

[2] Ates HC, Yetisen AK, Guder F (2021). Wearable devices for the detection of COVID-19. Nat. Electron..

[3] Zhang Y, Zhao P, Lu Q, Zhang Y, Lei H, Yu C, Yu J (2023). Functional additive manufacturing of large-size metastructure with efficient electromagnetic absorption and mechanical adaptation. Compos. A Appl. Sci. Manuf..

[4] Liu G (2021). Data collection in MI-assisted wireless powered underground sensor networks: Directions, recent advances, and challenges. IEEE Commun. Mag..

[5] Lu Z, Wu D, Ding H, Chen L (2021). Vibration isolation and energy harvesting integrated in a Stewart platform with high static and low dynamic stiffness. Appl. Math. Modell..

[6] Lu Z, Zhao L, Ding H, Chen L (2021). A dual-functional metamaterial for integrated vibration isolation and energy harvesting. J. Sound Vib..

[7] Fu S, Wu H, He W, Li Q, Shan C, Wang J, Hu C (2023). Conversion of dielectric surface effect into volume effect for high output energy. Adv. Mater..

[8] Rozgic D, Markovic D (2017). A miniaturized 0.78-mW/cm^2^ autonomous thermoelectric energy-harvesting platform for biomedical sensors. IEEE Trans. Biomed. Circuits Syst..

[9] Li T, Li Y (2023). Artificial intelligence for reducing the carbon emissions of 5G networks in China. Nat. Sustain..

[10] Jannat MKA, Islam MS, Yang S, Liu H (2023). Efficient Wi-Fi-based human activity recognition using adaptive antenna elimination. IEEE Access.

[11] Lyu T, Xu H, Zhang L, Han Z (2024). Source selection and resource allocation in wireless-powered relay networks: An adaptive dynamic programming-based approach. IEEE Internet Things J..

[12] Hou X, Xin L, Fu Y, Na Z, Gao G, Liu Y, Chen T (2023). A self-powered biomimetic mouse whisker sensor (BMWS) aiming at terrestrial and space objects perception. Nano Energy..

[13] Ouyang Y, Chappell WJ (2008). High frequency properties of electrotextiles for wearable antenna applications. IEEE Trans. Antennas Propag..

[14] So J, Thelen J, Qusba A, Hayes GJ, Lazzi G, Dickey MD (2009). Reversibly deformable and mechanically tunable fluidic antennas. Adv. Funct. Mater..

[15] Surender D, Khan T, Talukdar FA, Antar YMM (2021). Review on rectenna design and development strategies for wireless applications. IEEE Antennas Propag. Mag..

[16] Quddious A, Zahid S, Tahir FA, Antoniades MA, Vryonides P, Nikolaou S (2021). Dual-band compact rectenna for UHF and ISM wireless power transfer systems. IEEE Trans. Antennas Propag..

[17] Chen H, Wu H, Kan T, Zhang J, Li H (2023). Low-carbon economic dispatch of integrated energy system containing electric hydrogen production based on VMD-GRU short-term wind power prediction. Int. J. Electr. Power Energy Syst..

[18] *IEEE C95.1–2005-IEEE Standard for Safety Levels with Respect to Human Exposure to Radio Frequency Electromagnetic Fields, 3 kHz to 300 GHz*. https://standards.ieee.org/standard/C951-2005.html.

[19] Sanusi OM, Ghaffar FA, Shamim A, Vaseem M, Wang Y, Roy L (2019). Development of a 2.45 GHz antenna for flexible compact radiation dosimeter tags. IEEE Trans. Antennas Propag..

[20] Chen Y, Ku T (2016). A low-profile wearable antenna using a miniature high impedance surface for smartwatch applications. IEEE Antennas Wirel. Propag. Lett..

[21] An Z, Huang Y, Zhang R (2023). High-temperature multispectral stealth metastructure from the microwave-infrared compatible design. Compos. B Eng..

[22] Wang Q, Li P, Rocca P, Li R, Tan G, Hu N, Xu W (2023). Interval-based tolerance analysis method for petal reflector antenna with random surface and deployment errors. IEEE Trans. Antennas Propag..

[23] Yan S, Vandenbosch GAE (2016). Radiation pattern-reconfigurable wearable antenna based on metamaterial structure. IEEE Antennas Wirel. Propag. Lett..

[24] Haga N, Saito K, Takahashi M, Ito K (2009). Characteristics of cavity slot antenna for body-area networks. IEEE Trans. Antennas Propag..

[25] Rowe WST, Waterhouse RB (2003). Reduction of backward radiation for CPW fed aperture stacked patch antennas on small ground planes. IEEE Trans. Antennas Propag..

[26] Agarwal K, Guo YX, Salam B (2016). Wearable AMC backed near-endfire antenna for on-body communications on Latex substrate. IEEE Trans. Components Packag. Manuf. Tech..

[27] Ashyap AYI (2017). Compact and low-profile textile EBG-based antenna for wearable medical applications. IEEE Antennas Wirel. Propag. Lett..

[28] Gao GP, Yang C, Hu B, Zhang RF, Wang SF (2019). A wearable PIFA with an all-textile metasurface for 5GHz WBAN applications. IEEE Antenna Wirel. Propag. Lett..

[29] Singh N (2018). A dual band was rectifying antenna for RF energy harvesting. J Comput. Electron.

[30] Moro R, Agneessens S, Rogier H, Bozzi M (2012). Wearable textile antenna in substrate integrated waveguide technology. Electron. Lett..

[31] Sun H, Guo YX, He M, Zhong Z (2013). A dual-band rectenna using broadband Yagi antenna array for ambient RF power harvesting. IEEE Antennas Wireless Propag. Lett..

[32] *Dupont Kapton Polyimide Specification Sheet*. www.dupont.com/Kapton.

[33] Khaleel HR, Al-Rizzo HM, Rucker DG (2012). Compact polyimide-based antennas for flexible displays. J. Display Technol..

[34] Gao N, Liu J, Deng J, Chen D, Huang Q, Pan G (2024). Design and performance of ultra-broadband composite meta-absorber in the 200Hz–20kHz range. J. Sound Vib..

[35] Singh N, Kumar S, Kanaujia BK, Beg MT, Kumar S (2020). A compact broadband GFET based rectenna for RF energy harvesting applications. Microsyst. Technol..

[36] Singh N, Kumar S, Kanaujia BK, Beg MT, Kumar S (2020). A compact and efficient graphene FET based RF energy harvester for green communication. AEU Int. J. Electron. Commun..

[37] Singh N, Kanaujia BK, Beg MT, Khan T, Kumar S (2018). A dual polarized multiband rectenna for RF energy harvesting. AEU Int. J. Electron. Commun..

[38] Singh N, Kanaujia BK, Beg MT, Kumar S (2019). A triple band circularly polarized rectenna for RF energy harvesting. Electromagnetics.

[39] Awais Q, Jin Y, Chattha HT, Jamil M, Qiang H, Khawaja BA (2018). A compact rectenna system with high conversion efficiency for wireless energy harvesting. IEEE Access.

[40] Surender D, Halimi MA, Khan T, Talukdar FA, Antar YMM (2021). Circularly polarized DR-rectenna for 5G and Wi-Fi bands RF energy harvesting in smart city applications. IETE Tech. Rev..

[41] Surender D, Halimi MA, Khan T, Talukdar FA, Koul SK, Antar YMM (2021). 2.45 GHz Wi-Fi band operated circularly polarized rectenna for RF energy harvesting in smart city applications. J. Electromagn. Waves Appl..

[42] Wang Y, Chen P, Yong J, Xu W, Xu S, Liu K (2022). A comprehensive investigation on the selection of high-pass harmonic filters. IEEE Trans. Power Deliv..

[43] Huang X, Zhou L, Mao J (2019). Modified FSIW filter with N transmission zeros using BCB-based MEMS technology. IEEE Microwave Wirel. Components Lett..

[44] Huang X, Zhang X, Zhou L, Xu J, Mao J (2023). Low-loss self-packaged ka-band LTCC filter using artificial multimode SIW resonator. IEEE Trans. Circuits Syst. II Express Briefs.

[45] Huang X, Zhou L, Xu J, Zhang XY, Mao J (2022). BCB-based thin-film Ka-band quarter-mode SIW packaged filters with ultrawide stopband and independently controlled TZs. IEEE Trans. Microwave Theory Tech..

[46] Xu K (2021). Silicon electro-optic micro-modulator fabricated in standard CMOS technology as components for all silicon monolithic integrated optoelectronic systems. J. Micromech. Microeng..

[47] Lin W, Ziolkowski RW (2021). Electrically small, single-substrate huygens dipole rectenna for ultracompact wireless power transfer applications. IEEE Trans. Antennas Propag..

[48] Lin W, Ziolkowski RW (2020). Electrically small huygens CP rectenna with a driven loop element maximizes its wireless power transfer efficiency. IEEE Trans. Antennas Propag..

